# Prevention of Viral Hepatitis and HIV Infection among People Who Inject Drugs: A Systematic Review and Meta-Analysis

**DOI:** 10.3390/v16010142

**Published:** 2024-01-18

**Authors:** Yen-Ju Chen, Yu-Chen Lin, Meng-Tien Wu, Jenn-Yuan Kuo, Chun-Hsiang Wang

**Affiliations:** 1 Research Assistant Center, Tainan Municipal Hospital (Managed by Show Chwan Medical Care Corporation), Tainan 701033, Taiwan; 2a7101@tmh.org.tw (Y.-J.C.); 2a7102@tmh.org.tw (Y.-C.L.); 2a7103@tmh.org.tw (M.-T.W.); 2 Department of Food Nutrition, Chung Hwa University of Medical Technology, Tainan 717302, Taiwan; 3 Department of Hepatogastroenterology, Tainan Municipal Hospital (Managed by Show Chwan Medical Care Corporation), Tainan 701033, Taiwan

**Keywords:** prevention strategies, blood-borne virus infection, people who inject drugs (PWID), systematic review, meta-analysis

## Abstract

This study aimed to explore the current evidence on preventing blood-borne virus infections among people who inject drugs (PWID). We conducted a comprehensive search across three databases (PubMed, Embase, Cochrane Library) for relevant articles published in English between 2014 and 2023. We followed the Preferred Reporting Items for Systematic Reviews and Meta Analysis (PRISMA) guidelines, assessed the quality of the paper using the revised Cochrane Risk of Bias Tool (ROB 2), and conducted a meta-analysis using RevMan 5.3. Completing the harm reduction program (HRP) participation and receiving all three vaccine doses resulted in a 28% reduction in the risk of HBV infection (OR: 0.72, 95% CI: 0.37–1.42). Various interventions increased the willingness of PWIDs to undergo HCV treatment (OR: 5.91, 95% CI: 2.46–14.24) and promoted treatment adherence (OR: 15.04, 95% CI: 2.80–80.61). Taking PrEP, participating in HRP, and modifying risky behaviors were associated with a 33% reduction in the risk of HIV infection (OR: 0.67, 95% CI: 0.61–0.74). Conducting referrals, providing counseling, and implementing antiretroviral therapy resulted in a 44% reduction in the risk of viral transmission (OR: 0.56, 95% CI: 0.47–0.66). Co-infection may potentially compromise effectiveness, so it is important to consider drug resistance.

## 1. Introduction

The transmission of human immunodeficiency virus (HIV) continues to affect vulnerable populations, including heterosexual individuals, men who have sex with men (MSM), and people who inject drugs (PWID). Various biological, behavioral, and social factors influence the continued spread of HIV within these groups [1]. The development of antiretroviral therapy (ART) has transformed HIV/AIDS from a fatal disease into a manageable condition that can potentially be prevented [2]. However, as more individuals start ART, HIV drug resistance remains a growing threat to the long-term success of HIV treatment programs and epidemic control [3]. Combining viral phylogenetics with demographic and behavioral information provides valuable insights into the epidemiological processes that shape transmission networks at the population level [4,5,6]. Viral hepatitis, which includes hepatitis B virus (HBV) and hepatitis C virus (HCV), has a significant impact on public health. It leads to considerable morbidity and mortality due to chronic liver disease. HBV infections are primarily acquired during childbirth or in early childhood, while HCV infections are typically acquired in adulthood. Among individuals coinfected with HIV and HCV, the majority (59% of cases) are PWID [7]. Globally, it is estimated that there are 2.73 million people with HIV/HBV coinfection and 2.28 million people with HIV/HCV coinfection. Sub-Saharan Africa has the highest prevalence of HIV/HBV coinfection, accounting for 71% of all cases, while Eastern Europe and Central Asia have the highest prevalence of HIV/HCV coinfection, accounting for 27% of all cases [7]. The current strategy for testing viral hepatitis involves initial serological tests, which can be conducted using rapid diagnostic tests or laboratory-based immunoassays. Subsequently, virological testing is conducted to detect the presence of HBV DNA and HCV RNA, which guides treatment decisions. Furthermore, the estimated global prevalence of hepatitis B surface antigen (HbsAg) is 7.4% with an interquartile range (IQR) of 5.0–11.2%, and the seroprevalence of HCV antibody (anti-HCV) is 6.2% with an IQR of 3.4–11.9% in HIV-infected individuals [7].

During the past few decades, Taiwan has made significant progress in reducing the number of HBV infections through its comprehensive national public health policy, which includes universal vaccination against hepatitis B for newborns [8]. Recent advancements in the development of all-oral pan-genotypic treatments for HCV have achieved an impressive cure rate of over 90% [9]. Pre-exposure prophylaxis (PrEP) is a preventive strategy aimed at reducing HIV-1 transmission in high-risk populations. The evidence supports the safety and efficacy of oral PrEP in MSM, serodiscordant couples, and PWID [10]. Adherence to PrEP should be based on the risk of HIV transmission, whether through daily or event-driven (ED) dosing regimens. While a significant number of MSM showed strong adherence to PrEP in real-world situations, Taiwanese MSM who switched from daily to ED dosing regimens exhibited less consistent adherence. This suggests a requirement for innovative strategies to enhance PrEP adherence in this particular population [11].

The extensive use of multiple antiretroviral drugs often results In drug resistance due to the accumulation of mutations in the viral genome. Given the similar modes of transmission for HIV-1 and hepatitis viruses (e.g., HBV/HCV), primarily through contact with infected blood, administering combination drug therapy to patients co-infected with both HIV/HBV and HIV/HCV could potentially compromise effectiveness due to the emergence of drug resistance. Furthermore, prolonged use of such treatments could have adverse effects on liver function. To explore various intervention approaches aimed at reducing the incidence of blood-borne infectious diseases and review existing evidence for preventing viral hepatitis and HIV infections within this particular population. The study includes a comprehensive meta-analysis and a thorough literature review.

## 2. Materials and Methods

### 2.1. Search Strategy and Database

This study followed the guidelines of the preferred reporting items for systematic reviews and meta-analyses (PRISMA) [12,13,14] guidelines. We conducted a thorough search of three electronic databases, namely PubMed, Embase, and the Cochrane Library, to identify relevant articles. The search included all English-language publications from 1 January 2014 to 31 August 2023, without any geographical or article-type restrictions. In addition to the initial search results, we independently reviewed the reference lists of selected articles to identify any potentially overlooked studies. Our search used keywords related to the following topics: “viral hepatitis”, “HIV infections”, “substance abuse”, “prevention”, “interventions”, “HIV-positive”, “hepatitis B”, “hepatitis C”, “PWID”, “HBV vaccination”, “education and counseling”, “needle and syringe programs”, “risk reduction”, “harm reduction”, “co-infection”, “epidemiology”, “efficacy”, “adherence”, and “incidence” (please see Supplementary Table S1 for details). We exclusively considered studies published in English. The sociodemographic characteristics considered included: gender (male vs. female), age (under 25 years vs. 25 years and older), educational attainment (primary school or less vs. secondary school or higher education), monthly income (below minimum wage vs. minimum wage or above), and race (Asian vs. non-Asian). Our study included both experimental and observational quantitative research studies that had undergone peer review and were published in scholarly journals.

### 2.2. Inclusion and Exclusion Criteria

Inclusion criteria: The selected studies should focus on people who inject drugs (PWIDs) as the primary population of interest. These studies should provide details about interventions aimed at preventing infections caused by blood-borne viruses within this population. Additionally, the studies should include data on the prevalence rates of hBsAg, HCVAb, and HIV-1 seropositivity. Incidence and prevalence data should be linked to sociodemographic characteristics or established risk factors associated with viral infections. We specifically included randomized controlled trials (RCTs) or cluster-randomized trials (CRTs) in our criteria.

Exclusion criteria: Studies that did not involve PWIDs were not RCT or CRT, or were not published in a journal format, were excluded. Studies that did not provide clear descriptions of the diagnostic criteria for viral infections or did not use seropositive tests for diagnosis were also excluded. In cases where multiple studies used the same dataset, preference was given to those that provided more comprehensive information on the inclusion criteria.

### 2.3. Study Selection Process

The study selection process occurred in phases according to specific inclusion and exclusion criteria. Initially, two authors, Y.-J.C. and J.-Y.K., independently screened the titles and abstracts of articles, and they also individually reviewed the full articles of eligible studies. Any disagreements were resolved through consultation with a third author, C.-H.W. The authors Y.-J.C. and J.-Y.K. gathered data, such as the first author’s last name, year of publication, study design, and clinical information (e.g., hBsAb, hBsAg, HCVAb, HCV RNA, and HIV-1 viral load). They also collected data on intervention strategies, the total number of enrolled PWIDs in each study, the number of PWIDs testing positive for viral infections, the number of PWIDs participating in risk reduction programs, and the assessment of HBV/HCV/HIV-1 status based on sociodemographic characteristics and related risk factors. When necessary, the authors of the included studies were contacted for additional information. The second author Y.-C.L. randomly selected and cross-checked the extracted data.

### 2.4. Quality Assessment, Analysis, and PROSPERO Registry

Two authors, Y.-J.C and M.-T.W, evaluated the quality of the included papers using the updated Cochrane risk of bias tool for randomized trials (ROB 2) [15,16], which can be accessed at https://sites.google.com/site/riskofbiastool/ (accessed on 22 August 2023). This website also provides tools for evaluating non-RCTs of interventions and systematic reviews. The assessment process involved a series of prompting questions with potential responses: “yes”, “probably yes”, “probably no”, “no”, or “no information”. After evaluating all domains, an overall risk assessment result was determined and categorized as either “low risk of bias”, “some concerns”, or “high risk of bias”. It is important to note that we had previously decided not to exclude any studies based on their quality ratings.

For the meta-analysis, we used Review Manager Software version 5.3 (RevMan 5.3, The Cochrane Collaboration, Oxford, UK), which is available at http://tech.cochrane.org/revman accessed on 22 August 2023. We used the weighted mean difference and relative risk with 95% confidence intervals as summary statistics for continuous and dichotomous variables, respectively. Statistical results from the studies were combined using a suitable method for binomial data. Viral infection occurrences among different sociodemographic and known risk factor groups were compared using the relative risk, represented as the odds ratio (OR) in this study. To assess statistical heterogeneity, RevMan 5.3 was used, with additional evaluation using the I-squared statistic, which quantifies the proportion of variation in pooled estimates attributed to heterogeneity rather than chance. The selection of the meta-analysis model (fixed-effects or random-effects) was based on chi-squared tests and I-squared statistics to assess heterogeneity. Pooled effects were determined using the z-test. Forest plots were used to illustrate the effectiveness of various intervention measures. Subgroup analyses were conducted within the meta-analysis when selected studies showed significant heterogeneity. Potential publication bias was assessed using a funnel plot. A two-sided *p*-value of less than 0.05 was considered statistically significant.

**Figure 1:**
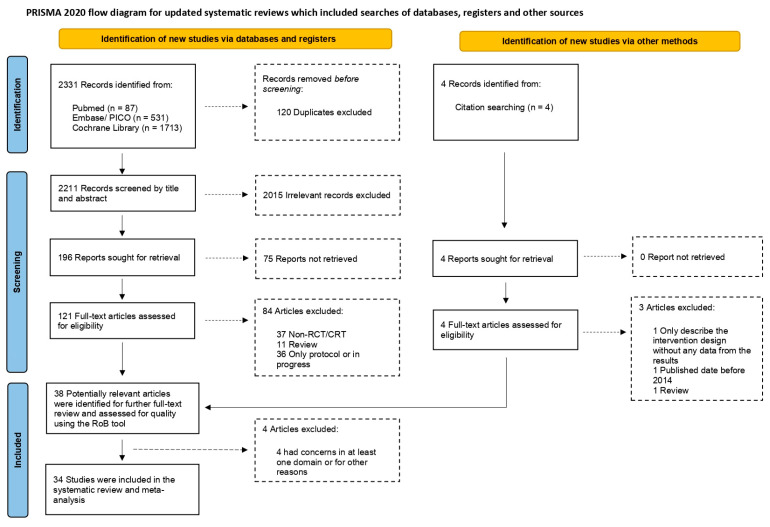
The flowchart illustrates the process of paper selection.

Furthermore, this study includes the Preferred Reporting Items for Systematic Reviews and Meta Analysis2020 flow diagram, Preferred Reporting Items for Systematic Reviews and Meta Analysis2020 checklist, and Preferred Reporting Items for Systematic Reviews and Meta Analysis2020 abstract checklist (please refer to the Supplementary Materials) and adheres to the ROB 2 guidance for paper selection evaluation. Relevant documents have also been registered with PROSPERO (CRD42023460728, registered on 16 September 2023). Initially, we identified 2331 citations, of which 121 published articles appeared relevant and were retrieved for further assessment. Among these, 84 were excluded for various reasons (see Figure 1). Ultimately, 38 eligible articles were included in this study, as detailed in Figure 1 and Tables S2 and S3. Detailed characteristics of the included RCTs and extracted endpoints are provided in Table S3. Finally, 34 trials were found to have a low risk of bias across all domains. 

## 3. Results

A thorough assessment of the efficacy and potential risks of different prevention strategies.

### 3.1. HBV Infection

**Figure 2:**
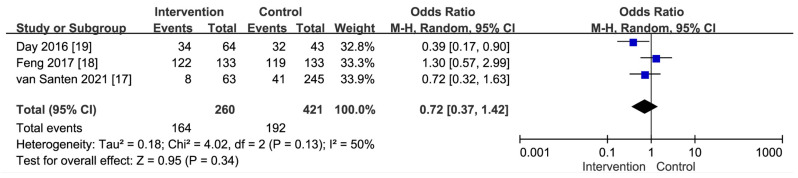
The combined seroconversion rate for HBV infection among PWIDs who completed HRP participation and received all three vaccine doses. The blue symbol represents the effectiveness of each study, while the black symbol shows the overall effectiveness of this subgroup [17,18,19].

Taking HBV as an example, prevention strategies involve the early implementation of harm-reduction programs (HRP) [17] and ensuring the completion of vaccination [18,19,20,21]. The primary outcome focused on comparing seroconversion rates between the intervention and control groups. Completing the HRP participation and receiving all three vaccine doses led to a 28% reduction in the risk of HBV infection (odds ratio: 0.72, 95% CI: 0.37–1.42, *p*-value = 0.34) (refer to Figure 2).

### 3.2. HCV Infection

The primary mode of HCV transmission is through the use of injection drugs. Treating PWID is essential for the elimination of HCV, as curative therapy will not only reduce the burden of disease over time but also interrupt onward transmission. The main outcome compares the success rate of treatment (sustained virologic response, SVR) in each treatment group. Secondary analyses involve evaluating adherence, reinfection rates, and viral resistance to treatment. Because PWIDs have a high lifetime risk of incarceration, prisons are important settings for engaging and treating individuals living with hepatitis C. Additionally, strategies for linking individuals to hepatitis C care after community re-entry are also crucial for prevention.

**Figure 3:**
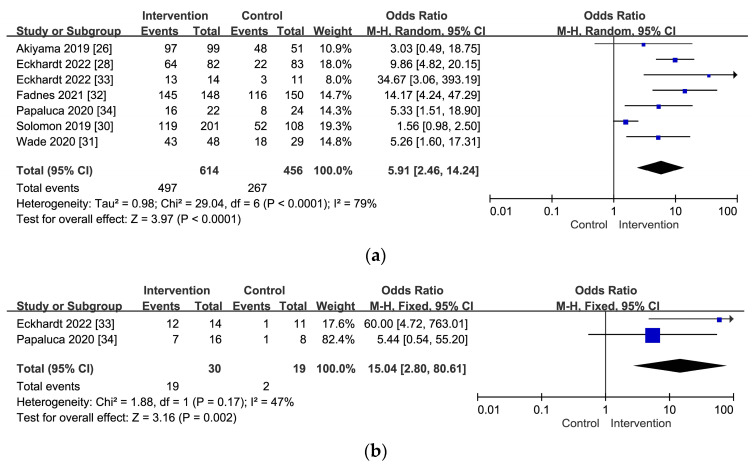

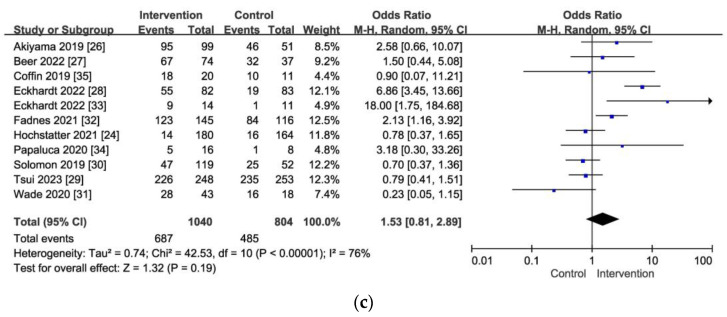
(**a**) The overall time-to-treatment initiation for PWIDs with a positive HCV RNA report for HCV infection [26,28,30,31,32,33,34]. (**b**) The overall treatment completion rate for HCV infection among PWIDs who underwent drug therapies [33,34]. (**c**) The overall sustained virologic response (SVR) rate for HCV infection among PWIDs who completed treatment [24,26,27,28,29,30,31,32,33,34,35].

Through multiple interventions such as peer outreach [22], mobile health [23,24], financial incentives [25], onsite HCV care [17,26,27,28,29], and vertically integrated HCV testing [30,31,32,33,34,35], the willingness of PWIDs in the intervention group to undergo HCV treatment is significantly enhanced (OR: 5.91, 95% CI: 2.46–14.24, *p*-value < 0.0001) (Figure 3a). These interventions also promote treatment adherence (OR: 15.04, 95% CI: 2.80–80.61, *p*-value = 0.0002) (Figure 3b) and increase the likelihood of achieving an HCV cure (OR: 1.53, 95% CI: 0.81–2.89, *p*-value = 0.19) (Figure 3c) compared to the control group.

### 3.3. HIV Infection

Social networks offer potential opportunities for behavior change among high-risk populations. To prevent the continued spread of HIV among PWIDs, strategies can be discussed in two parts: (1) For PWIDs who are not infected with HIV, enhancing their understanding of HIV, the importance of self-protection, and regular screening can be achieved through peer-based education, harm reduction programs, needle and syringe exchange initiatives, and health consultations. (2) Individuals who have tested seropositive for HIV through screening should not only be referred for medical confirmation but should also receive intensified health and educational consultations to encourage them to follow their doctor’s instructions and use ART regularly to effectively suppress viral replication. Furthermore, uninfected sexual partners should regularly take PrEP to reduce the risk of viral transmission.

**Figure 4:**
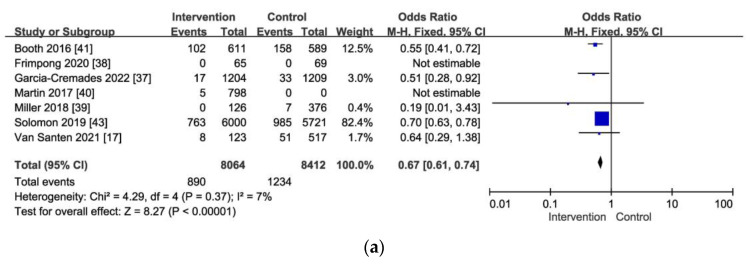

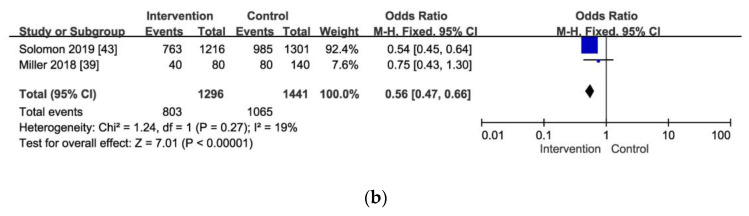
(**a**) The combined seroconversion (i.e., new diagnoses) for HIV infection among PWIDs who took PrEP, completed HRP participation, and modified their risky HIV behaviors [17,37,38,39,40,41,43]. (**b**) The studies examine the combined viral suppression of HIV infection among PWIDs who receive HIV support through referrals, counseling, and ART use [39,43].

In this study, 12 RCTs were included for further analysis. Among them, 8 studies [17,36,37,38,39,40,41,42,43] focused on HIV-negative PWIDs and aimed to improve access to and adherence to PrEP and ART. Participating in the HRP led to a significant reduction in HIV risk. Furthermore, providing combined rapid HIV and HCV testing with immediate on-site results increased awareness of viral infections. The common result among these studies was HIV seroconversion. As shown in Figure 4a, the overall result indicated a 33% reduction in the risk of HIV infection (OR: 0.67, 95% CI: 0.61–0.74, *p*-value < 0.00001).

Three additional studies [39,43,44] focused on HIV-positive PWIDs. The aim of these studies, conducted through referrals and counseling, was to improve patients’ adherence to ART and effectively suppress viral replication, while also modifying their risky behaviors to reduce the risk of viral transmission. In two of these studies, the primary outcome was viral suppression. The overall result showed a 44% reduction in the risk of HIV infection (OR: 0.56, 95% CI: 0.47–0.66, *p*-value < 0.00001) (Figure 4b).

## 4. Discussion and Conclusions

PWIDs face an increased risk of various health issues, both infectious and non-infectious, which can have serious implications for their overall health and lifespan. Approaching these individuals without judgment and engaging in open discussions about their current drug use can provide valuable insights into their readiness for addiction treatment and help identify modifiable risk factors associated with complications arising from PWIDs. It is crucial to routinely screen all PWID for HIV infection, latent tuberculosis, hepatitis B, and hepatitis C. Furthermore, it is important to administer vaccinations for hepatitis B, tetanus, and pneumonia when necessary. Another essential preventive measure is to offer pre-exposure prophylaxis (PrEP) for HIV infection to PWID. Prisons [34,45], drug rehabilitation centers [18,25,40], and methadone clinics [17,20,32,38,46] are crucial facilities for identifying, treating, and preventing viral hepatitis infections among PWIDs. However, low adherence to treatment is a widespread issue that reduces the individual and public benefits of various health interventions. Financial incentives improved compliance with HBV vaccination and ensured that these vaccinations were administered within specified appointment times [19,20]. Four RCTs specifically examined contingency management aimed at HBV vaccinations [18,19,20,21]. Despite their small sample sizes, one study found that contingency management, which involved monetary incentives, was significantly more effective than standard care in achieving completion of HBV vaccination within 6 months (87% vs. 66%) [19]. Complete vaccination with three doses of the vaccine can elicit a strong immune response, regardless of whether the full dose is administered each time. The high-level response generated by high-dose vaccination may result in longer-lasting protection [18].

Injecting drugs is a significant transmission route for HCV and HIV. Factors such as seeking financial support or drugs through sexual activities, as well as employment status, increase the probability of sharing needles or syringes. Homelessness and incarceration further increase the risk of needle/syringe sharing [36]. Drug addiction can impede adherence to ART medication. The low adherence to ART among methamphetamine users may be attributed to the combined effects of methamphetamine and HIV on the brain, resulting in impaired neurocognitive function. The use of short- and long-acting opioids can also impact physiological and behavioral functions. Consequently, using multiple drugs may decrease the likelihood of future ART initiation among PWIDs [44]. When modeling the probability of HIV infection over time in high-risk seronegative participants receiving a placebo, serodiscordant heterosexual partners (partners taking PrEP) showed the lowest risk of HIV infection [37]. Providing on-site rapid bundled testing for HIV and HCV resulted in a higher self-reported receipt of test results for both conditions. This has the potential to improve engagement in prevention services, encourage timely treatment initiation, and ultimately reduce the morbidity and mortality associated with HIV and HCV [30,38]. Furthermore, interventional measures can also reduce the risk of HIV transmission to injection partners [39]. Methadone maintenance treatment (MMT) is an effective strategy for managing opioid use disorder and preventing overdose. Ensuring that incarcerated individuals have continuous access to MMT has a long-lasting and positive impact on various opioid-related outcomes after their release [45]. PWIDs with a history of heroin use or incarceration were more likely to choose to take PrEP. This suggests that participants based their decision to take PrEP, at least partially, on their perceived risk of acquiring HIV infection [40]. The evidence indicates that incorporating peer education into comprehensive HIV prevention programs for PWID could improve access to and adherence to PrEP and ART [41]. Moreover, it is challenging to develop behavioral interventions among PWIDs that can ensure sustained communication about HIV prevention topics over time [47].

The incidence of HCV is highest during the first 3–5 years of an individual’s injection drug use. A 12-week course of direct-acting antivirals (DAAs) for HCV typically leads to a cure, irrespective of HIV status. However, patents and high prices have created barriers to access for individuals living with HCV, especially among PWIDs. Insufficient access to and coverage of harm reduction interventions contribute to the intersecting epidemics of HIV and HCV. Consequently, the highest prevalence of HCV is observed among PWIDs, who face additional challenges to treatment, including stigma, discrimination, and other structural barriers. Riskier injection practices, such as sharing and reusing equipment, were linked to treatment failure [29]. Financial incentives were implemented to improve HCV treatment attendance among PWIDs; however, it is essential to assess their long-term effectiveness [25]. Given the ongoing risky behavior observed in this population, future research should investigate targeted interventions to prevent HCV reinfection. Rapid intervention with same-day prescription and dispensation of pan-genotypic DAA therapy was feasible. It resulted in significantly higher rates of treatment initiation, completion, and cure compared to the standard of care [31,33]. Regions with a high prevalence of PWID which drives blood-borne virus transmission, may consider offering testing and treatment through local treatment programs [23,27,28,32]. As mobile health innovations continue to emerge and evolve, the opportunity to address complex, comorbid health conditions on a larger scale is expanding [24]. Consistently maintaining adequate total dose adherence throughout HCV treatment is important for achieving SVR among PWIDs [26,46,48].

This study aimed to provide a comprehensive assessment of blood-borne infection prevention strategies tailored to PWIDs. The significance of ART in HIV management and disease prevention is emphasized. Furthermore, the effectiveness of PrEP in preventing hepatitis B is highlighted. Other strategies, such as education and counseling, needle, and syringe exchange programs, and promoting condom use, are discussed in the context of preventing viral hepatitis infections. However, it is important to recognize that their impact may be limited when drug resistance is a factor. As indicated in the introduction that the biggest burden of disease is in Africa. However, no studies from the African continent were included in the review. This could be due to the lack of RCTs in PWIDs in Africa or a relatively lower burden on use of injectable drugs in Africa. Limitations of this study include the intensive nature and multiple financial components of the intervention, which may potentially restrict its implementation in other regions. Additionally, because the intervention included multiple components, we cannot confidently determine which specific age group contributed to the high treatment rates achieved. Ensuring access to ART and PrEP for individuals with HIV drug resistance, as well as their sexual partners, is essential in reducing the prevalence of viral hepatitis infections. Further research is necessary to identify and develop targeted prevention strategies tailored to this specific population. Addressing these challenges can ultimately improve the overall health outcomes of HIV patients with drug resistance and contribute to the reduction of viral hepatitis transmission.

## Data Availability

The data presented in this study is available in Section 2.4 and Supplementary Materials.

## Supplementary Materials

The following supporting information can be downloaded at: https://www.mdpi.com/article/10.3390/v16010142/s1, Figure S1: PRISMA 2020 for abstracts checklist, Figure S2: PRISMA 2020 checklist, Table S1: Search strategy for PubMed and related databases, Table S2: Excluded studies and reasons, and Table S3: Details for extracting data from included randomized controlled trials (RCTs) and cluster-randomized trials (CRTs).
