# Supplementary material for: Prevention of Viral Hepatitis and HIV Infection among People Who Inject Drugs: A Systematic Review and Meta-Analysis
Source: Viruses. 2024 Jan 18;16(1):142. doi: 10.3390/v16010142 (PMC10820947; doi:10.3390/v16010142)
Supplement: Supplementary file 1 [file viruses-16-00142-s001.zip › viruses-2761833-Supplementary.pdf]

Supplementary materials

PRISMA 2020 for Abstracts Checklist

Section and Topic	Item #	Checklist item	Reported (Yes/No)
**TITLE**			
Title	1	Identify the report as a systematic review.	Yes
**BACKGROUND**			
Objectives	2	Provide an explicit statement of the main objective(s) or question(s) the review addresses.	Yes
**METHODS**			
Eligibility criteria	3	Specify the inclusion and exclusion criteria for the review.	Yes
Information sources	4	Specify the information sources (e.g. databases, registers) used to identify studies and the date when eachwas last searched.	Yes
Risk of bias	5	Specify the methods used to assess risk of bias in the included studies.	Yes
Synthesis of results	6	Specify the methods used to present and synthesise results.	Yes
**RESULTS**			
Included studies	7	Give the total number of included studies and participants and summarise relevant characteristics of studies.	Yes
Synthesis of results	8	Present results for main outcomes, preferably indicating the number of included studies and participants for each. If meta-analysis was done, report the summary estimate and confidence/credible interval. If comparing groups, indicate the direction of the effect (i.e. which group is favoured).	Yes
**DISCUSSION**			
Limitations of evidence	9	Provide a brief summary of the limitations of the evidence included in the review (e.g. study risk of bias,inconsistency and imprecision).	Yes
Interpretation	10	Provide a general interpretation of the results and important implications.	Yes
**OTHER**			
Funding	11	Specify the primary source of funding for the review.	Yes
Registration	12	Provide the register name and registration number.	Yes

From: Page MJ, McKenzie JE, Bossuyt PM, Boutron I, Hoffmann TC, Mulrow CD, et al. The PRISMA 2020 statement: an updated guideline for reporting systematicreviews. BMJ 2021;372:n71. doi: 10.1136/bmj.n71

For more information, visit: http://www.prisma-statement.org/

Figure S1: PRISMA 2020 for abstracts checklist

PRISMA 2020 Checklist

Section and Topic	Item #	Checklist item	Location where item is reported
**TITLE**			
Title	1	Identify the report as a systematic review.	Title
**ABSTRACT**			
Abstract	2	See the PRISMA 2020 for Abstracts checklist.	Abstract
**INTRODUCTION**			
Rationale	3	Describe the rationale for the review in the context of existing knowledge.	Introduction (Section 1)
Objectives	4	Provide an explicit statement of the objective(s) or question(s) the review addresses.	Introduction (Section 1)
**METHODS**			
Eligibility criteria	5	Specify the inclusion and exclusion criteria for the review and how studies were grouped for the syntheses.	Materials and Methods (Section 2.2)
Information sources	6	Specify all databases, registers, websites, organisations, reference lists and other sources searched or consulted to identify studies. Specify the date when each source was last searched or consulted.	Materials and Methods (Section 2.1)
Search strategy	7	Present the full search strategies for all databases, registers and websites, including any filters and limits used.	Materials and Methods (Section 2.1; Table S1)
Selection process	8	Specify the methods used to decide whether a study met the inclusion criteria of the review, including how many reviewers screened each record and each report retrieved, whether they worked independently, and if applicable, details of automation tools used in the process.	Materials and Methods (Section 2.3)
Data collection process	9	Specify the methods used to collect data from reports, including how many reviewers collected data from each report, whether they worked independently, any processes for obtaining or confirming data from study investigators, and if applicable, details of automation tools used in the process.	Materials and Methods (Section 2.3)
Data items	10a	List and define all outcomes for which data were sought. Specify whether all results that were compatible with each outcome domain in each study were sought (e.g. for all measures, time points, analyses), and if not, the methods used to decide which results to collect.	Materials and Methods (Section 2.1)
	10b	List and define all other variables for which data were sought (e.g. participant and intervention characteristics, funding sources). Describe any assumptions made about any missing or unclear information.	Materials and Methods (Sections 2.2 & 2.3)
Study risk of bias assessment	11	Specify the methods used to assess risk of bias in the included studies, including details of the tool(s) used, how many reviewers assessed each study and whether they worked independently, and if applicable, details of automation tools used in the process.	Materials and Methods (Section 2.4)
Effect measures	12	Specify for each outcome the effect measure(s) (e.g. risk ratio, mean difference) used in the synthesis or presentation of results.	Materials and Methods (Section 2.4)
Synthesis methods	13a	Describe the processes used to decide which studies were eligible for each synthesis (e.g. tabulating the study intervention characteristics and comparing against the planned groups for each synthesis (item #5)).	Materials and Methods (Section 2.4)
	13b	Describe any methods required to prepare the data for presentation or synthesis, such as handling of missing summary statistics, or data conversions.	Materials and Methods (Section 2.4)

PRISMA 2020 Checklist

Section and Topic	Item #	Checklist item	Location where item is reported
	13c	Describe any methods used to tabulate or visually display results of individual studies and syntheses.	Materials and Methods (Section 2.4)
	13d	Describe any methods used to synthesize results and provide a rationale for the choice(s). If meta-analysis was performed, describe the model(s), method(s) to identify the presence and extent of statistical heterogeneity, and software package(s) used.	Materials and Methods (Section 2.4)
	13e	Describe any methods used to explore possible causes of heterogeneity among study results (e.g. subgroup analysis, meta-regression).	Materials and Methods (Section 2.4)
	13f	Describe any sensitivity analyses conducted to assess robustness of the synthesized results.	Materials and Methods (Section 2.4)
Reporting bias assessment	14	Describe any methods used to assess risk of bias due to missing results in a synthesis (arising from reporting biases).	Materials and Methods (Section 2.4)
Certainty assessment	15	Describe any methods used to assess certainty (or confidence) in the body of evidence for an outcome.	Materials and Methods (Section 2.4)
**RESULTS**			
Study selection	16a	Describe the results of the search and selection process, from the number of records identified in the search to the number of studies included in the review, ideally using a flow diagram.	Figure 1
	16b	Cite studies that might appear to meet the inclusion criteria, but which were excluded, and explain why they were excluded.	Supplementary materials (Table S2)
Study characteristics	17	Cite each included study and present its characteristics.	Supplementary materials (Table S3)
Risk of bias in studies	18	Present assessments of risk of bias for each included study.	Materials and Methods (Section 2.4)
Results of individual studies	19	For all outcomes, present, for each study: (a) summary statistics for each group (where appropriate) and (b) an effect estimate and its precision (e.g. confidence/credible interval), ideally using structured tables or plots.	Results (Figures 2 - 4)
Results of syntheses	20a	For each synthesis, briefly summarise the characteristics and risk of bias among contributing studies.	Results & Section 2.4
	20b	Present results of all statistical syntheses conducted. If meta-analysis was done, present for each the summary estimate and its precision (e.g. confidence/credible interval) and measures of statistical heterogeneity. If comparing groups, describe the direction of the effect.	Results (Figures 2 - 4)
	20c	Present results of all investigations of possible causes of heterogeneity among study results.	Results (Section 3)

PRISMA 2020 Checklist

Section and Topic	Item #	Checklist item	Location where item is reported
	20d	Present results of all sensitivity analyses conducted to assess the robustness of the synthesized results.	Results (Section 3)
Reporting biases	21	Present assessments of risk of bias due to missing results (arising from reporting biases) for each synthesis assessed.	NA
Certainty of evidence	22	Present assessments of certainty (or confidence) in the body of evidence for each outcome assessed.	Results (Section 3)
**DISCUSSION**			
Discussion	23a	Provide a general interpretation of the results in the context of other evidence.	Discussion and conclusion (Section 4)
	23b	Discuss any limitations of the evidence included in the review.	Discussion and conclusion (Section 4)
	23c	Discuss any limitations of the review processes used.	Discussion and conclusion (Section 4)
	23d	Discuss implications of the results for practice, policy, and future research.	Discussion and conclusion (Section 4)
**OTHER INFORMATION**			
Registration and protocol	24a	Provide registration information for the review, including register name and registration number, or state that the review was not registered.	Section 2.4; Yen-Ju Chen; PROSPERO (no. CRD42023460728)
	24b	Indicate where the review protocol can be accessed, or state that a protocol was not prepared.	Section 2.4
	24c	Describe and explain any amendments to information provided at registration or in the protocol.	Section 2.4
Support	25	Describe sources of financial or non-financial support for the review, and the role of the funders or sponsors in the review.	Funding
Competing interests	26	Declare any competing interests of review authors.	Not applicable
Availability of data, code and other materials	27	Report which of the following are publicly available and where they can be found: template data collection forms; data extracted from included studies; data used for all analyses; analytic code; any other materials used in the review.	Section 2.4 and Supplementary Materials

Figure S2: PRISMA 2020 checklist

Table S1. Search strategy for PubMed and related databases

	Term 1: Viruses	"Hepatitis B Virus" OR "HBV" OR "Hep B" OR "Hepatitis C" OR "Hepatitis C Virus" OR "HCV" OR "Hep C" OR "Hepatitis C" OR "Human immunodeficiency virus" OR "HIV" OR "HIV-1"
AND	Term 2: Populations	"Injection drug users" OR "injecting drug users" OR "persons who inject drugs" OR "people who inject drugs" OR "PWID" OR "substance abuse"
AND	Term 3: Interventions	"HBV vaccination" OR "education and counseling" OR "needle and syringe programs" OR "NSPs" OR "syringe services programs" OR "SSPs" OR "syringe exchange programs" OR "SEPs" OR "needle exchange programs" OR "NEPs" OR "harm reduction" OR "preexposure prophylaxis" OR "pre-exposure prophylaxis" OR "PrEP" OR "condom usage" OR "community support" OR "social support"
AND	Term 4: Outcomes	"Risk reduction" OR "co-infection" OR "efficacy" OR "adherence"
AND	Term 5: Diagnosis	"Screening" OR "testing" OR "prevalence" OR "incidence" OR "positivity" OR "seropositive" OR "positive"

1. PubMed / Query box

Search: (((("Hepatitis B Virus" OR "HBV" OR "Hep B" OR "Hepatitis C" OR "Hepatitis C Virus" OR "HCV" OR "Hep C" OR "Hepatitis C" OR "Human immunodeficiency virus" OR "HIV" OR "HIV-1"[MeSH Terms]) AND ("Injection drug users" OR "injecting drug users" OR "persons who inject drugs" OR "people who inject drugs" OR "PWID" OR "substance abuse"[MeSH Terms])) AND ("HBV vaccination" OR "education and counseling" OR "needle and syringe programs" OR "NSPs" OR "syringe services programs" OR "SSPs" OR "syringe exchange programs" OR "SEPs" OR "needle exchange programs" OR "NEPs" OR "harm reduction" OR "preexposure prophylaxis" OR "pre-exposure prophylaxis" OR "PrEP" OR "condom usage" OR "community support" OR "social support"[MeSH Terms])) AND ("Risk reduction" OR "co-infection" OR "efficacy" OR "adherence"[MeSH Terms])) AND ("Screening" OR "testing" OR "prevalence" OR "incidence" OR "positivity" OR "seropositive" OR "positive"[MeSH Terms])

Filters: In the last 10 years, English (n=87)

Sort by: Journal

2. Embase / PICO

#1 'injection drug user'/exp AND ('hepatitis vaccine'/exp OR 'medical education'/exp OR 'preventive health service'/exp OR 'syringe exchange program'/exp OR 'harm reduction'/exp OR 'preexposure prophylaxis'/exp OR 'condom use'/exp OR 'community support'/exp OR 'social support'/exp) AND

('risk reduction'/exp OR 'coinfection'/exp OR 'therapy'/exp OR 'patient compliance'/exp OR 'screening'/exp OR 'prevalence'/exp OR 'incidence'/exp OR 'seropositive reaction'/exp)

#2 AND ('acquired immune deficiency syndrome'/dm OR 'acute hepatitis c'/dm OR 'chronic hepatitis b'/dm OR 'chronic hepatitis c'/dm OR 'drug dependence'/dm OR 'hepatitis b'/dm OR 'hepatitis c'/dm OR 'heroin dependence'/dm OR 'human immunodeficiency virus infection'/dm OR 'infection rate'/dm OR 'reinfection'/dm OR 'virus hepatitis'/dm)

#3 AND (2014:py OR 2015:py OR 2016:py OR 2017:py OR 2018:py OR 2019:py OR 2020:py OR 2021:py OR 2022:py OR 2023:py)

n = 531

3. Cochrane Library

Topics: Infectious disease

Year: 2014 - 2023

Language: English

matching people who inject drugs in Title Abstract Keyword OR Injecting drug users in Title Abstract Keyword OR "injecting-drug users" in Title Abstract Keyword OR Injection drug users in Title Abstract Keyword OR persons who inject drugs in Title Abstract Keyword - (Word variations have been searched)

n = 1,713

Table S2. Excluded studies and reasons

Citations	Reasons
Heo, M.; Pericot-Valverde, I.; Niu, J.; Norton, B. L.; Akiyama, M. J.; Nahvi, S.; Arnsten, J. H.; Litwin, A. H., More intensive hepatitis C virus care models promote adherence among people who inject drugs with active drug use: the PREVAIL Study. *Journal of viral hepatitis* **2022**.	The study has identified concerns regarding this result in at least one domain.
Heo, M.; Pericot-Valverde, I.; Rennert, L.; Akiyama, M. J.; Norton, B. L.; Gormley, M.; Agyemang, L.; Arnsten, J. H.; Litwin, A. H., Hepatitis C Virus Direct-Acting Antiviral Treatment Adherence Patterns and Sustained Viral Response Among People Who Inject Drugs Treated in Opioid Agonist Therapy Programs. *Clinical infectious diseases* **2021,** 73, (11), 2093-2100.	The study has identified concerns regarding this result in at least one domain.
Zhang, J. Y.; Li, Z. B.; Zhang, L.; Wang, J.; Huang, L. P.; Zhan, G. L.; Li, Z.; Du, J.; Zhao, M., DOES IT WORK? -a randomized controlled trial to test the efficacy of HCV and HIV-related education on drug users in MMT, China. *BMC infectious diseases* **2019,** 19, (1), 774.	The study has identified concerns regarding this result in at least one domain.
Li, L.; Hien, N. T.; Liang, L. J.; Lin, C.; Lan, C. W.; Lee, S. J.; Tuan, N. A.; Tuan, L. A.; Thanh, D. C.; Ha, N. T. T., Efficacy of Communication Training of Community Health Workers on Service Delivery to	Although the study had a low risk of bias, we excluded it because its focus was not aligned with our article.

People Who Inject Drugs in Vietnam: a Clustered Randomized Trial. *American journal of public health* **2018,** 108, (6), 791-798.	

Table S3. Details for extracting data from included randomized controlled trials (RCTs) and cluster-randomized trials (CRTs)

Viral types	First author & Year	Sample size	Randomized groups	Related risk factors	Outcomes	Detection times
**HBV**						
1	van Santen 2021 [17]	N = 308 (HIV/HCV/HBV-negative)<br><br>**Netherlands**	Complete harm reduction program (HRP), partial HRP, and no HRP	Injection frequency, and needle and syringe program (NSP) coverage	Seroconversion	Baseline, per 4 – 6 months assessments
2	Feng 2017 [18]	N = 480 (aged 18 – 70 years, HBsAg anti-HBs and HIV negative)<br><br>**China**	IM 20, IM 60, and control	High-level response rate	Seroconversion	Baseline, 6-, 7-, and 12-months assessments
3	Day 2016 [19]	N = 201 (aged 16 years or older, non-HBV infection, ≤ 1 HBV vaccination dose, unknown infection & vaccination status)<br><br>**Australia**	Incentive and standard of care (SOC)	Age and vaccination series completion	Seroconversion and vaccination completion	Baseline, 12 weeks post-completed vaccination
4	Weaver 2014 [20]	N = 210 (aged 18 – 65 years, non-HBV infection, and w/o vaccination experiences)	Escalating value contingency management, fixed value contingency management, and treatment as usual	Gender, race, employment, prison history, and drug use	Attendance and vaccination completion	Within 7-, 28-days, and 3 months of the first vaccination (day 0)

		**UK**				
5	Bowman 2014 [21]	N = 595 (aged 18 years or older, non-HBV infection, and w/o vaccination experiences)<br><br>**USA**	Accelerated HBV vaccination schedule and standard schedule	Accelerated treatment, age, race, gender, employment, less healthy, and main source of syringes past 3 months	Vaccination completion	Baseline, 1-, 2-, 6-, and 7-months assessments
**HCV**						
1	Tsui 2023 [29]	N = 501 (aged 18-70 years, HCV-infected, and not treated with direct-acting antivirals)<br><br>**USA**	Modified directly observed therapy (mDOT) and patient navigator (PN)	Age, living situation, and drug substance	Injection practices and sustained virologic response (SVR)	Baseline, end of treatment, 12-, 24-, 36-, 48-, and 60-weeks post-treatment
2	Marshall 2022 [25]	N = 593 (aged 18 years or older, currently not receiving HCV treatment)<br><br>**Australia**	Financial incentives and unspecified	Being Aboriginal or Torres Strait Islander, unemployed, completion of secondary school, and mainly injected heroin in month prior	Hepatitis C RNA-positive	Onsite HCV RNA Testing
3	Eckhardt 2022 [33]	N = 47 (aged 18 – 29 years, HCV-infected, and treatment-naive)	Rapid treatment and usual care	Race, insurance, and drug injection	HCV RNA testing and SVR	Baseline, day 2 – 7, treatment initiation, and 12-month assessments

		**USA**				
4	Beer 2022 [27]	N = 129 (aged 18 – 70 years, HCV-infected, and not co-infected with HBV or HIV)<br><br>**Scotland**	DOT, fortnightly provision, and fortnightly provision w/ psychological intervention	Gender, source of income, and injecting history	Treatment adherence and cure rate (SVR_{12})	Baseline and 12 weeks post-treatment
5	Eckhardt 2022 [28]	N = 167 (aged 18 years or older, HCV RNA-positive, and treatment-naive)<br><br>**USA**	Accessible care and usual care	Incarceration history	SVR and re-infection	Baseline, 3-, 6-, 9-, and 12-months assessments
6	van Santen 2021 [17]	N = 137 (HIV/HCV/HBV-negative)<br><br>**Netherlands**	Complete HRP, partial HRP, and no HRP	Injection frequency, and needle and syringe program (NSP) coverage	Seroconversion	Baseline, per 4 – 6 months assessments
7	Hochstatter 2021 [24]	N = 416 (aged 18 years or older)<br><br>**USA**	Receive medication-assisted treatment (MAT) only, and MAT+A-CHESS	Shared injection equipment	HCV testing and SVR_{12}	Baseline, 4-, 8-, 12-, 16-, 20-, and 24-months assessments
8	Fadnes 2021 [32]	N = 298 (chronic HCV infection)<br><br>**Norway**	Integrated treatment and treatment as usual	Gender, education, Sources of income, and substance use last 30 days	Time-to-treatment initiation, SVR_{12}, and HCV Ab test	Every 4 weeks, 12 weeks post-treatment

9	Wade 2020 [31]	N = 136 (age, HCV-infection, treatment-naïve, and not co-infected with HBV or HIV)<br><br>**Australia & New Zealand**	Primer care and local hospital (SOC)	Gender, employment, accommodation, and OST history	SVR_{12} and treatment uptake rate	Baseline, 12 weeks post-treatment
10	Papaluca 2020 [34]	N = 46 (aged 18 – 65 years, active HCV infection, and treatment-naive)<br><br>**Australia**	Care navigation and SOC	Gender, race, and drug injection	Commenced DAA, completion of treatment, and SVR_{12}	Baseline, released within 6 months, and 12 weeks post-treatment
11	Kronfli 2020 [48]	N = 78 (aged 18 years or older, male inmates, and w/o HCV test experiences)<br><br>**Canada**	OraQuick and venipuncture	Accepted HCV screening rate	HCV Ab test and acceptability	Baseline, 10 weeks assessments
12	Hochstatter 2020 [23]	N = 235 (aged 18 years or older)<br><br>**USA**	Hep-Net and control	Gender, race, education level, employed, and substance use	Substance use reduction, overdose prevention, and HCV testing	Baseline, 3-, and 6-months post-enrollment
13	Frimpong 2020 [38]	N = 162 (aged 18 years or older, HCV/HIV status unknown or negative)	Treatment and SOC	Education, and unprotected intercourse in the past month	HIV/HCV testing	Baseline, 1 month after randomization

		**USA**				
14	Broad 2020 [22]	N = 380 (aged 18 years or older, HCV Ab status unknown)<br><br>**Canada**	Point-to-case (POC) test and treating-as-usual (TAU)	Negative healthcare provider experience (past year), frequency of injection drug use, has a primary healthcare provider, and housing status	HCV Ab testing and engagement in HCV care	Baseline
15	Solomon 2019 [30]	N = 11721 (aged 18 years or older)<br><br>**India**	Integrated care centers (ICC) intervention and usual care	Gender, education, income, alcohol use, even shared needle/syringe, drugs injected in prior 6 months, and even use needle exchange program	HCV prevalence, self-reported HCV testing history, and SVR	Baseline, evaluation (2 years later)
16	Coffin 2019 [35]	N = 31 (aged 18 years or older, active HCV, genotype 1)<br><br>**USA**	mDOT and unobserved	Health status, even shared syringes	HIV/HCV testing, SVR_{12}, and reinfection rate	Baseline, 2-, 8- (end of treatment), 12-, and 36-weeks post-treatment
17	Akiyama 2019 [26]	N = 150 (aged 18 years or older, had HCV genotype 1, treatment-naive)	DOT, group treatment (GT), and self-administered	Daily/window timeframe adherence, psychiatric illness,	HCV RNA test, adherence, treatment completion, and SVR_{12}	Baseline, 4-, 8-, 12-weeks treatment, and 4-, 12-, 24-weeks post-treatment

		**USA**	individual treatment (SIT)	drinking alcohol to intoxication		
**HIV**						
1	Van Sluytman 2022 [36]	N = 696 (aged 18 years or older, 232 HIV-negative & 464 network members) **USA**	Low-risk, paraphernalia risk, and high sex/moderate risk	Age and money/drugs for sex	HIV support and risk networks	Baseline, and 6-month follow-up
2	Ha 2022 [44]	N = 502 (aged 18 – 45 years, HIV-infected) **Indonesia, Ukraine, and Vietnam**	The integrated intervention of supported ART and SOC	Numbers of injected/non-injected other drugs in last 3 months, days injected drugs last month, CD_4 count at screening, and study site	ART acceptance and adherence	Baseline, 26-, and 52-weeks visits
3	Garcia-Cremades 2022 [37]	N = 177 (aged 20 – 60 years, HIV-negative) **Thailand**	Daily oral Tenofovir (TDF) and daily oral placebo	Gender and drug injection frequency	Seroconversion (i.e., new diagnoses)	Baseline, per 3 months assessments
4	van Santen 2021 [17]	N = 640 (HIV/HCV/HBV-negative) **Netherlands**	Complete HRP, partial HRP, and no HRP	Injection frequency, and needle and syringe program (NSP) coverage	Seroconversion (i.e., new diagnoses)	Baseline, per 4 – 6 months assessments

5	Frimpong 2020 [38]	N = 162 (aged 18 years or older, HCV/HIV status unknown or negative)<br><br>**USA**	Treatment and SOC	Education and unprotected intercourse in the past month	HIV/HCV testing (i.e., new diagnoses)	Baseline, 1 month after randomization
6	Solomon 2019 [43]	N = 11721 (aged 18 years or older)<br><br>**India**	Integrated care centers (ICC) intervention and usual care	Gender, education, income, alcohol use, even shared needle/syringe, drugs injected in prior 6 months, and even use needle exchange program	Self-reported HIV testing, HIV prevalence, seroconversion, viral suppression, and effectiveness	Baseline and evaluation (2 years later)
7	Brinkley-Rubinstein 2018 [45]	N = 179 (aged 18 years or older, incarcerated, and had engaged in methadone-maintenance treatment (MMT)<br><br>**USA**	MMT continuation group and SOC	Duration of incarceration (days), drug use at 12 months (prior 30 days), and HIV risk behaviors (prior 30 days)	Engagement in MMT, re-arrest, re-incarceration, fatal and non-fatal overdose, emergency department usage, substance use, and HIV risk behaviors	1-, 6-, and 12-months post-release
8	Miller 2018 [39]	N = 502 (aged 18 – 60 years, HIV-infected) and 806 injection partners (HIV-negative)	The integrated intervention of supported ART and SOC	Little access to ART and medication-assisted treatment (MAT)	ART acceptance, ART use, HIV incidence, and viral suppression	Baseline and 12 – 24 months followed up

		**Indonesia, Ukraine, and Vietnam**				
9	Martin 2017 [40]	N = 1315 (aged 20 – 60 years, HIV-negative)<br><br>**Thailand**	TDF and placebo	Age, injected heroin, and had incarcerated experiences	HIV incidence, PrEP uptake, and PrEP adherence	Baseline, per 3 months assessments
10	Booth 2016 [41]	N = 1200 (aged 16 years or older, HIV-negative)<br><br>**Ukraine**	Testing and counseling block plus social network intervention block, and testing and counseling block	Age, daily injector, mean years of injecting, mean log injection frequency, front and back loading with others, front and back loading with dealer/others/both, shared works, and more than one sexual partner	Reducing drug and sex risk behaviors and seroconversion	Baseline, 6-, and 12-months assessments
11	Mihailovic 2015 [47]	N = 227 (aged 18 years or older)<br><br>**USA**	Experimental and control	Education, needle sharing, and condom use	HIV testing and the effect of interventions on the change in frequency of conversation	Baseline, 6-, 12-, and 18-months assessments
12	Mackesy-Amiti 2014 [42]	N = 854 (aged 15 – 30 years, HIV/HCV-negative)	Peer education intervention (PEI) and video-discussion control	Race, homeless past 6 months, binge alcohol weekly, number of sex partners, and	Effect of intervention on the change in sexual risk behavior	Baseline, 3-, and 6-months assessments

		**USA**		casual male partners		
